# Effect of Microhydration on the Temporary Anion States of Pyrene

**DOI:** 10.1021/acs.jpclett.2c00523

**Published:** 2022-04-14

**Authors:** Aude Lietard, Jan R. R. Verlet

**Affiliations:** Department of Chemistry, Durham University, Durham DH1 3LE, United Kingdom

## Abstract

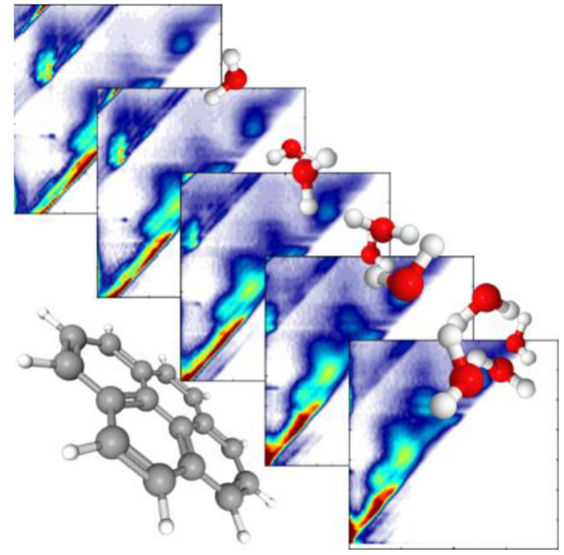

The influence of incremental hydration (≤4) on the electronic resonances of the pyrene anion is studied using two-dimensional photoelectron spectroscopy. The photoexcitation energies of the resonances do not change; therefore, from the anion’s perspective, the resonances remain the same, but from the neutral’s perspective of the electron–molecule reaction, the resonances decrease in energy by the binding energy of the water molecules. The autodetachment of the resonances shows that hydration has very little effect, showing that even the dynamics of most of the resonances are not impacted by hydration. Two specific resonances do show changes that are explained by the closing of specific autodetachment channels. The lowest-energy resonance leads to efficient electron capture as observed through thermionic emission and evaporation of water molecules (dissociative electron attachment). The implications of low-energy electron capture in dense molecular interstellar clouds are discussed.

Pyrene (Py, C_16_H_10_) is a one of the simplest two-dimensional polycyclic aromatic hydrocarbon (PAH) molecules consisting of four fused benzene rings, as shown in the inset of Figure 1. Its optical and electronic properties have lent themselves to numerous technological uses.^1−3^ Py and PAHs in general are also commonly found in differing natural environments ranging from pyrolytic^4^ to cold interstellar molecular clouds.^5−7^ In the latter, PAHs can act as a reservoir of precursor molecules for the formation of complex organic molecules through reactions that take place in isolation or on ice grains.^8−12^ However, dense molecular clouds are generally cold (tens of kelvin).^12^ For a reaction to proceed, it should be either barrierless or driven by external factors such as ultraviolet (UV) radiation or electrons. Despite the opacity of the cloud, much laboratory work has been done to understand the interaction of UV radiation with PAHs in the presence of ice.^13−20^ On the contrary, there has been much less work devoted to understanding how PAHs interact with very low-energy electrons, especially at the molecular level and in the presence of water molecules. For example, the low-lying resonances of Py^–^ have only recently been measured and assigned.^21^ The resonances in PAH are important not only as potential sources of reactive molecular species but also because models predict that PAHs are the dominant carrier of negative charge in the clouds rather than electrons,^22−25^ which requires the capture of free electrons through resonances. These models assume that only PAHs containing more than 30 C atoms (*n*_C_), as these are deemed to have a sufficiently large electron affinity, are effective at capturing the electron; therefore, only large PAHs carry the negative charge, while smaller PAHs, such as Py, are not considered to be capable of capturing free electrons. This conclusion is in agreement with our previous study of the resonances of the isolated Py^–^.^21^ However, what if Py is not isolated as would be the case in ice grains; can solvent molecules mediate the electron capture for smaller PAHs? Here, we aim to address this question by using two-dimensional (2D) photoelectron spectroscopy of the corresponding solvated anion clusters and show that the addition of just a few water molecules can lead to the formation of ground state Py^–^.

**Figure 1:**
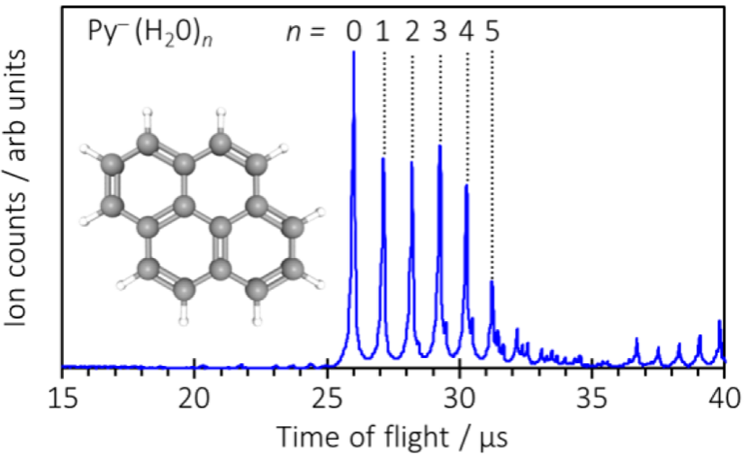
Mass spectrum of Py^–^(H_2_O)_*n*_ with the structure of pyrene inset.

Reactions between a free electron and a neutral target molecule are mediated by resonances that form temporary negative ions.^26−28^ As these resonances lie in the electronic continuum, autodetachment is an open channel. However, as autodetachment is not instantaneous, nuclear dynamics can take place on the resonance surface leading to internal conversion^29^ and formation of stable anionic ground state molecules. For an isolated system, the total energy remains above the detachment threshold and electrons can be emitted statistically from the anion ground state through thermionic emission,^30−32^ which has a distinctive spectral profile peaking at zero electron kinetic energy (eKE). In general, the spectral profile of the autodetached electrons can offer much insight into the resonance dynamics as this effectively probes the electron energy loss spectra.^29,33−35^ Here, we probe the resonances of Py^–^(H_2_O)_*n*_ using 2D photoelectron spectroscopy. In this, mass-selected anions (Figure 1) are exposed to optical radiation (laser pulses with a duration of ∼5 ns), which detaches the electron to produce a photoelectron spectrum. This process is repeated many times at various photon energies, *hv*, that span the electron detachment continuum. These together form a 2D map of the evolution of the photoelectron spectra as a function of *hv*.^34^

Figure 2 shows the 2D photoelectron spectra for Py^–^(H_2_O)_*n*_ with 0 ≤ *n* ≤ 4. The 2D spectra are composed of photoelectron spectra that have been recorded over the ranges of 1.0 eV ≤ *hv* ≤ 4.5 eV for *n* = 0–3 and 1.1 eV ≤ *hv* ≤ 4.5 eV for *n* = 4. The *hv* increment between spectra was 0.05 eV. Figure 2 is composed of a total of 365 photoelectron spectra (taken from their respective photoelectron images).

**Figure 2:**
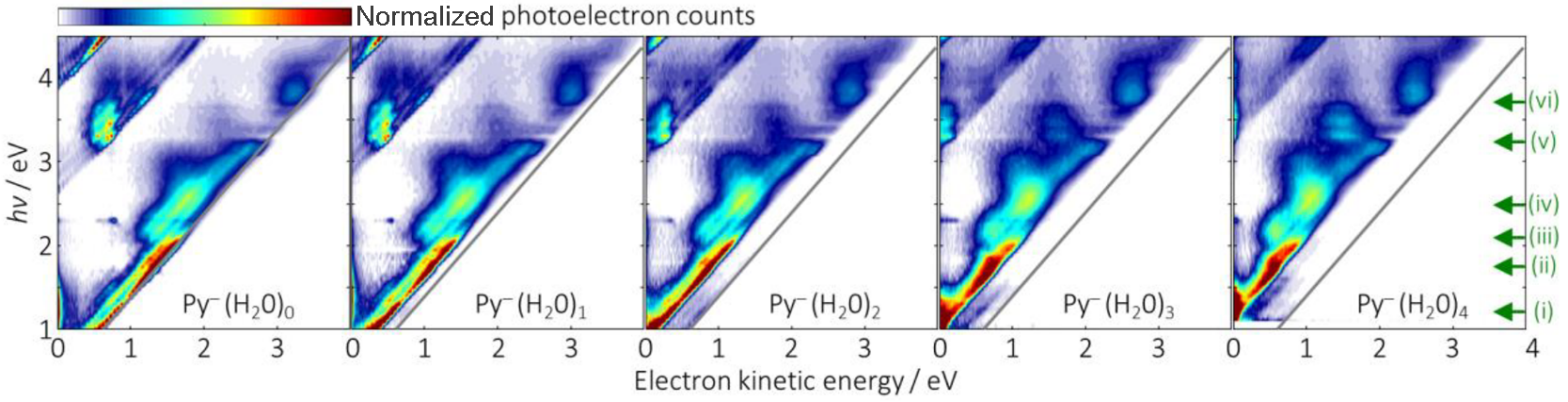
2D photoelectron spectra of Py^–^(H_2_O)_*n*_ for *n* = 0–4. The gray diagonal line shows the adiabatic detachment energy of the bare Py^–^. On the right, left-pointing green arrows indicate the locations of resonances (i) ^2^B_1u_, (ii) (1)^2^B_3g_, (iii) ^2^B_2g_, (iv) ^2^B_2g_(1p2h), and (v) (2)^2^B_3g_. Resonance vi has not been assigned.

We have previously presented the 2D photoelectron spectrum of Py^–^.^21^ In that work, we highlighted the different features and provided a detailed computational study to enable us to assign the observed electronic resonances in this range.^21^ The vertical (excitation) energies of the resonances are indicated on the right of Figure 2 by left-pointing arrows and are labeled (i)–(vi). Symmetry labels (*D*_2*h*_) are defined with Py lying in the *x–y* plane with *y* along the long axis. In order of increasing energy, the resonances correspond to the ^2^B_1u_, (1)^2^B_3g_, ^2^B_2g_, ^2^B_2g_(1p2h), and (2)^2^B_3g_, and the final resonance vi remains unassigned. The 1p2h label denotes that this resonance has mostly one-particle, two-hole character. The impact of the resonances on the 2D photoelectron spectrum can be clearly seen for most resonances. The ^2^B_1u_ resonance (i) leads to photoelectrons peaking at zero kinetic energy (i.e., thermionic emission). Resonance ^2^B_2g_ (iii) leads to a change in the apparent Franck–Condon profile with electrons being emitted with a lower kinetic energy compared to direct detachment to the continuum. Resonance ^2^B_2g_(1p2h) (iv) again has a changing Franck–Condon profile and leads to electrons with a constant kinetic energy as *hv* increases. The (2)^2^B_3g_ resonance (v) leads to an abrupt change in the preferred final state, switching from the neutral ground state for lower-energy resonances to the first excited (triplet) state for this resonance. This reverts for the resonance labeled (vi). Trends associated with the onset of resonances can also be clearly identified in the corresponding angular distributions of the emitted electrons (see the Supporting Information). While the above spectral changes and profiles hold much information about the dynamics of the resonances, as discussed elsewhere,^34−39^ the focus of the present study is on the evolution of these changes (and therefore the dynamics of the resonances) as water molecules are clustered onto Py^–^.

The addition of water molecules incrementally increases the adiabatic detachment energy (ADE). The gray diagonal line shows the ADE of Py^–^, and this is reproduced for the 2D photoelectron spectra of Py^–^(H_2_O)_*n*_ with 1 ≤ *n* ≤ 4 for comparison. This increase in ADE results from the energy of the binding of the water molecules to Py^–^: as the water–anion binding energy is greater than the water–neutral binding energy, higher photon energies are required to access the neutral surface. However, the most striking aspect of Figure 2 is that the positions of the resonances in terms of excitation energies do not appear to change with an increase in the degree of hydration. One can easily draw horizontal lines across Figure 2 to demonstrate that the location of the resonances does not change. A particularly clear example of this is the (2)^2^B_3g_ resonance (v), where the excitation energy is invariant with hydration, including the vibrational fine structure. This invariance is also present for all of the other resonances that can be identified [in the 2D photoelectron spectra and in their associated angular distributions (see the Supporting Information)]. In addition to the invariance of the excitation energy to hydration, the spectral profiles of the autodetachment from the various resonances do not change significantly for most resonances. This therefore suggests that the dynamics (in terms of both lifetimes and decay mechanisms) of most of the resonances are not affected by the presence of the water molecules. Apparently, the solvent acts as a simple spectator. The ^2^B_1u_ resonance and the (2)^2^B_3g_ resonance do show changes in spectral profiles, and these are discussed next.

In Figure 3, the energies of the relevant states of Py^–^(H_2_O)_*n*_ are plotted as a function of *n*. These energies are referenced to that of the neutral ground ^1^A_g_ state of Py(H_2_O)_*n*_. The decrease in the energy of the ground ^2^A_u_ state of Py^–^(H_2_O)_*n*_ reflects the increase in binding energy as mentioned above. All resonances are parallel to the ground anion state because the excitation energies are invariant with *n*. Also shown is the first excited ^3^B_2u_ state of the neutral Py, which can be clearly identified in Figure 2. The energy of this state as a function of *n* is parallel to the neutral ground state.

**Figure 3:**
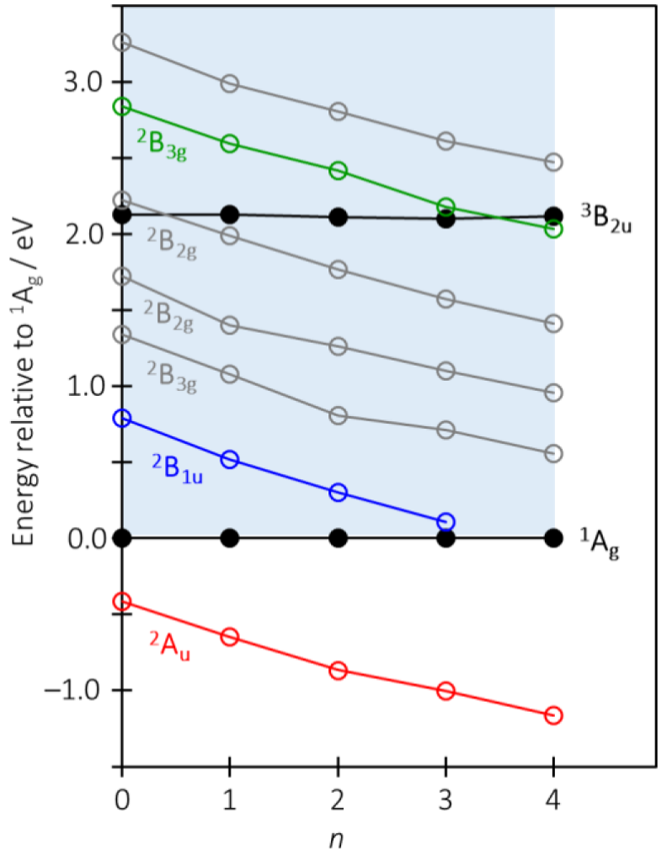
Energy of the states of neutral and anionic Py as a function of hydration, *n*. The energies are shown relative to the energy of the neutral ground ^1^A_g_ state. Data for neutral states are shown as black filled circles. Data for anionic states are shown as empty circles. The red line shows the data for the anionic ground state. The blue and green circles show the data for the ^2^B_1u_ and (2)^2^B_3g_ resonances that are further discussed in the text.

The graphing of the energy relative to that of the neutral ground state in Figure 3 effectively allows us to view the resonances from the perspective of an electron impacting the neutral molecule.^40^ In other words, as *n* increases, the kinetic energy required to excite a resonance decreases. Take, for example, the lowest-energy ^2^B_1u_ resonance, which lies 0.80 eV above the neutral ground state (its excitation energy is 1.21 eV in Py^–^, and the ADE of Py is 0.41 eV^21,41^). The addition of one H_2_O molecule increases the ADE by 0.24 eV, so that the resonance now lies 0.56 eV above the neutral. Additional H_2_O molecules lead to a further decrease in the resonance energy. This ^2^B_1u_ resonance and the (2)^2^B_3g_ resonance are highlighted specifically in Figure 3 because they are also the two resonances for which some changes can be seen in the 2D photoelectron spectra with an increase in *n*, which are discussed below.

The ^2^B_1u_ resonance shows that some population decays by thermionic emission as evidenced by the very low eKE peak in the 2D photoelectron spectrum of Py^–^. Because the ^2^B_1u_ resonance is the lowest excited state for Py^–^, the presence of thermionic emission requires that the ground electronic state is re-formed. The addition of one H_2_O molecule does not much affect the 2D spectrum except for increasing the ADE. However, closer inspection shows that there is a small contribution at a higher eKE than possible from detachment from Py^–^(H_2_O)_1_. This is shown more clearly in Figure 4, where the photoelectron spectra taken at an *hv* of 1.20 eV are compared for the different clusters. The dominant peak of Py^–^(H_2_O)_1_ at an eKE of ∼0.6 eV corresponds to direct detachment; however, there is also a peak at an eKE of ∼0.8 eV, which has the same energy and profile as the detachment peak from bare Py^–^. The peak is generated by the excitation to the ^2^B_1u_ resonance of Py^–^(H_2_O)_1_, which subsequently decays by internal conversion to access the ground electronic state. The total energy of the system now is still ∼0.5 eV above the neutral ground state, so the cluster can undergo unimolecular decay. This can be in the form of thermionic emission (which is seen in Figures 2 and 4) and through loss of a water molecule. The latter the leaves the dehydrated Py^–^, which can absorb an additional photon to produce the photoelectron spectrum of Py^–^ as shown in Figure 4. This process is analogous to dissociative electron attachment, which is well-known to produce stable anions following electron attachment. For Py^–^(H_2_O)_2_, the same evaporation process can take place, and Figure 4 clearly shows two peaks that correlate with the loss of one and two H_2_O molecules. Interestingly, the apparent thermionic emission has decreased, although caution should be used in comparing the intensities of the various signals because we have no information about the time scales of the electron or H_2_O evaporation. For Py^–^(H_2_O)_3_ and Py^–^(H_2_O)_4_, similar H_2_O evaporation is observed with the loss of up to three and four water molecules, respectively. Hence, by evaporative cooling, stable anions can be readily formed following population of the lowest-energy resonance in Py.

**Figure 4:**
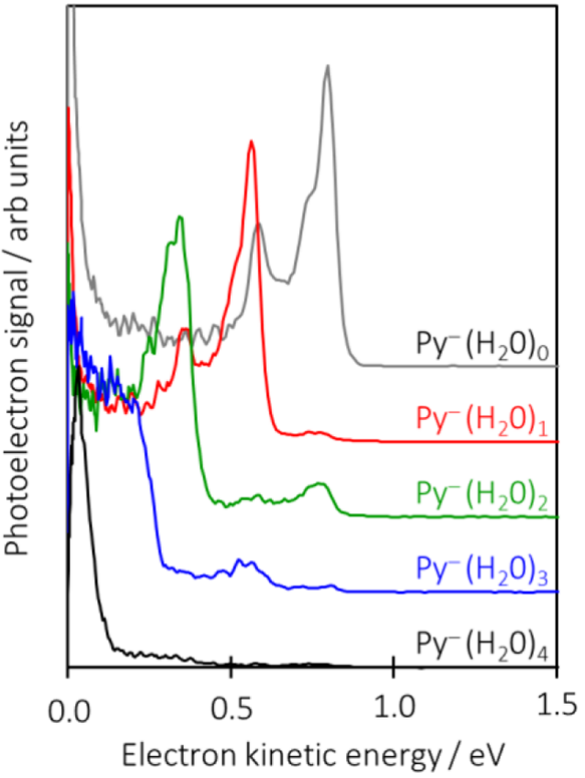
Photoelectron spectra of Py^–^(H_2_O)_*n*_ taken at an *hv* of 1.2 eV, highlighting that peaks arise from the photodetachment of dehydrated clusters following excitation to the ^2^B_1u_ resonance.

The other resonance highlighted in Figure 3 that shows changes in the photoelectron spectra as the degree of hydrating increases is due to the (2)^2^B_3g_ resonance, (v). This resonance is very noticeable in the 2D photoelectron spectrum of Py^–^ because of its preference to decay to the ^3^B_2u_ state of the neutral. As *n* increases, however, the relative signal leaving the neutral in its ground ^1^A_g_ state increases. This is most apparent for Py^–^(H_2_O)_*n*≥2_. The reason for the change in preference for the final state can be understood by reference to Figure 3. The energy of the (2)^2^B_3g_ resonance decreases relative to that of the neutral states as *n* increases, and when *n* = 4, the energy of the (2)^2^B_3g_ resonance lies below the ^3^B_2u_ state of the neutral. In other words, as increases, the decay channel to leave the neutral in the ^3^B_2u_ state closes at *n* = 4 and the only decay route is by autodetachment to the ^1^A_g_ state. The evolution is not abrupt but rather gradual, which suggests that the rate of autodetachment decreases as the energy of the resonance approaches the ^3^B_2u_ threshold, which likely is a manifestation of a centrifugal barrier to autodetachment from the (2)^2^B_3g_ resonance.^42^

In both highlighted cases of the ^2^B_1u_ (i) and (2)^2^B_3g_ resonances (v), the obvious changes in dynamics can be correlated by the closing of specific detachment channels. From Figure 3, the ^2^B_1u_ resonance in Py^–^(H_2_O)_4_ is essentially degenerate with the neutral ground state, so from the perspective of electron attachment at very low energies, this resonance can serve as a doorway resonance for the formation of stable anions by subsequent evaporative cooling by losing just a single water molecule.^40,43^ Hence, in relation to the electron capture of low-energy electrons by PAHs in dense molecular clouds, small PAHs (Py has 16 C atoms) can also capture electrons very effectively if just a few solvent molecules are present, which would be the case in ice grains. Note that our observation that the solvent is practically a spectator suggests that the nature of the solvent might not be important. This again is relevant to ice grains in which H_2_O is thought to be the most abundant,^8,10^ but other species such as MeOH, CO, CO_2_, NH_3_, etc., are also present. Our work extends our recent study on anthracene, and two of its N-substituted derivatives, acridine and phenazine, in which we similarly demonstrated that resonances do not shift from the perspective of the anion but do from the perspective of an electron impacting a neutral system.^40^ It is worth noting though that the molecules studied thus far using 2D photoelectron spectroscopy are weakly interacting with H_2_O, and one might expect the water to form a cluster above a plane of the Py^–^.^44^ In that case, the photoexcited states of the anion might be expected to be unaffected by the water cluster as the transition dipole moments lie in the plane of pyrene. Some changes in excitation energy with hydration might be anticipated for more strongly interacting molecules. We certainly suspect that dynamics might change in more strongly interacting cases as implicated in electron impact studies.^45−48^ Here, however, it is remarkable that the autodetachment dynamics from the resonances do not change significantly with solvation except when the resonances approach neutral thresholds.

In conclusion, we have shown that the temporary anion resonances in pyrene–water clusters stabilize to the same extent as the ground state of the anion does as hydration increases. The decay dynamics of the resonance, however, is not affected by the presence of water except in cases in which the resonance approaches or crosses a neutral threshold. For the lowest-energy resonance, this leads to ground state formation with the subsequent evaporation of water molecules (i.e., dissociative electron attachment), leaving a stable pyrene anion. From an interstellar perspective, this suggests that relatively small PAHs such as pyrene can be effective sinks for free electrons in dense molecular clouds if solvent molecules are condensed on the PAH.

## Methods

The experiment has been described in detail elsewhere, and only the details are given here.^49^ Pyrene anions were produced using a heated (230 °C) pulsed valve to produce a molecular beam (3 bar Ar backing gas), which was crossed near the throat of the expansion by an electron beam (500 μA, 300 eV). Water was introduced by adding a drop of liquid water to the backing line. Anions were extracted in a time-of-flight mass spectrometer. Its focus coincided with the center of a velocity map imaging photoelectron spectrometer, where mass-selected Py^–^(H_2_O)_*n*_ ions were intersected with light from a Nd:YAG-pumped optical parametric oscillator (∼5 ns pulses, 10 Hz). The liberated photoelectrons were detected and raw photoelectron images acquired at each wavelength. Photoelectron spectra and angular distributions were extracted from the raw images using polar onion peeling.^50^ The resulting spectra were calibrated using the known spectrum of iodide and have a resolution of ∼3% of the electron kinetic energy.

## Supporting Information Available

2D photoelectron angular distributions quantified by the β_2_ anisotropy parameter (PDF)
Transparent Peer Review report available (PDF)
