# Supplementary material for: Effect of Microhydration on the Temporary Anion States of Pyrene
Source: J Phys Chem Lett. 2022 Apr 14;13(16):3529–33. doi: 10.1021/acs.jpclett.2c00523 (PMC9084602; doi:10.1021/acs.jpclett.2c00523)
Supplement: Supplementary file 1 — jz2c00523_si_001.pdf [file jz2c00523_si_001.pdf]

Supporting Information for:

Effect of Micro-Hydration on the Temporary Anion States of Pyrene

Aude Lietard and Jan R. R. Verlet*

Department of Chemistry, Durham University, Durham DH1 3LE, United Kingdom

*j.r.r.verlet@durham.ac.uk

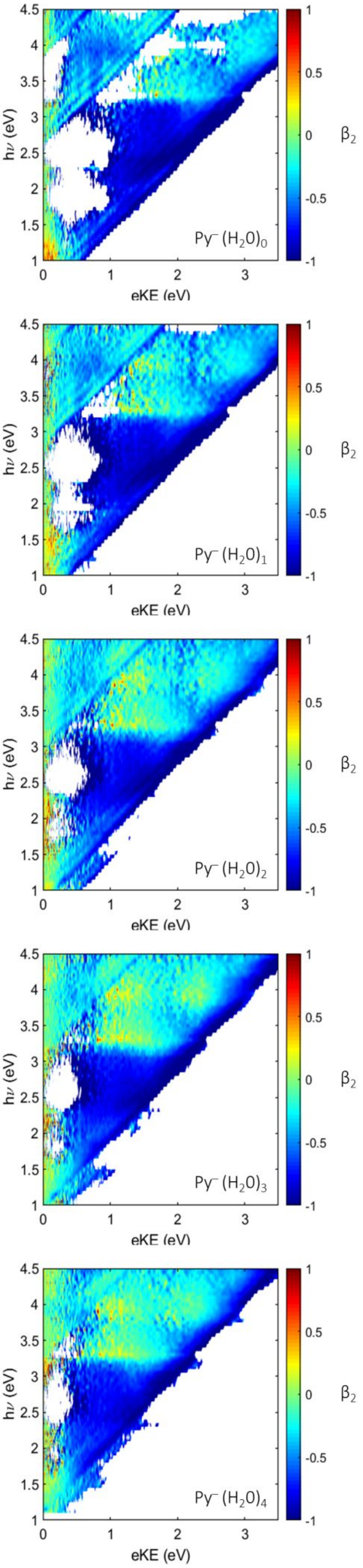

Figure S1: 2D photoelectron β_2 spectra of Py⁻(H$_2$O)$_n$ for n = 0 – 4.
