# Supplementary material for: Effect of Microhydration on the Temporary Anion States of Pyrene
Source: J Phys Chem Lett. 2022 Apr 14;13(16):3529–33. doi: 10.1021/acs.jpclett.2c00523 (PMC9084602; doi:10.1021/acs.jpclett.2c00523)
Supplement: Supplementary file 2 — jz2c00523_si_002.pdf [file jz2c00523_si_002.pdf]

jz-2022-00523y.R1

Name: Peer Review Information for "Effect of Micro-Hydration on the Temporary Anion States of Pyrene"

First Round of Reviewer Comments

Reviewer: 1

Comments to the Author
I offer the following suggestions to improve this manuscript in the context of fully supporting its publication in JPCL:

0) The water evaporation process described here is really analogous to dissociative electron attachment which is well-known to produce stable anion from electron attachment. I think that this should be pointed out explicitly to help frame this work for those interested in the formation of anion in the interstellar medium.

1) On page 3, line 33, it should refer to Figure 1 instead of Figure 2.

2) The authors gloss over the description of Figure 2 as if everybody was able to understand them by now. It might be just me making an early descent into senility, but even after seeing these 2D plots multiple times, I really struggle with how to interpret them. This is even worst here since each panel is very small. I understand that they were stacked horizontally to emphasize that the resonances don't change in energy with hydration, but it took me an entire (American-sized) cup of coffee to figure it out. It might be useful to dress these figure a bit to help the non-experts.

3) Referring to the anion resonances strictly by their symmetry is confusing at times since there are two 2B3g and two 2B2g. Maybe a letter in from of the state? X2Au, a2B1u, etc.

4) Some description of the expected interaction of water with the pyrene anion would be useful. I assume that they form weakly interacting clusters on top of the plane of the pyrene, similar to the naphtalene-water in ref 44? In that case, it might be too surprising that the stabilization is similar for all the pyrene anion excited-states (which all have transition dipoles moment in the molecular plane?).

5) In Figure 4, I don't see the loss of 3-4 waters for $Py(H_2O)_4$ mentioned in the text. (I squinted and stared really hard).

6) As a side comment, the detection of the water loss relies on the dissociation and photodetachment within the same laser pulse which might really understate the importance of this pathway relative to autodetachment/thermionic emission. Using a secondary MS stage to separate to the photofragments, as typically used in ion action spectroscopy, would enable the direct measurement of the branching ratio for the evaporation process.

Reviewer: 2

Comments to the Author
This is another nice 2D photoelectron spectroscopic study from the Verlet group. It is rather similar to their recent Nature chemistry (13/2021/737) opus which applied similar techniques to

anthracene and nitrogen substituted anthracene derivative, which also reported similar findings. However, the current work focuses on pyrene and solvated pyrene molecules.
The main findings are that resonances in the solvated pyrene molecules become stabilized by solvation (with respect to electron loss) but have very similar energies relative to the anion ground state regardless of the level of solvation. The results also highlight the possibility of relatively small PAHs acting as negative charge carriers in the interstellar media. The results are sufficiently different to the authors' previous work (pyrenes vs anthracenes), sufficiently novel and definitely interesting enough to warrant publication in JPCLett.
The paper is very accessible/readable and I enjoyed the narrative very much.

There are some minor typos that the authors might want to address, but scientifically I have very little to add, although the authors may want to consider backing up the statements on pages 7/8 regarding the two photon process producing Py-. Have the authors attempted to observe the power scaling of the photon intensity dependence?

Minor corrections:
Page 2 line 10 "have lent" rather than "have lend"
Page 2, line 22 "10s of K" rather than "10s K"
Page 3, line 31 "using 2D" rather than "we use 2D"
Page 6, line 17 "referenced" rather than "reference"
Page 7, line 17 "molecules lead to" rather than "molecules leads to"
Page 8, line 50 "hydration increases is due to the" rather than "hydrating increases is the"

Reviewer: 3

Comments to the Author
1. What is the major advance reported in the paper?
This is an experimental study on the photodetachment from the mixed pyrene-water anions. The anions are mass selected and thus the authors have full control on the hydration degree. The main experimental observation – that the resonance energies are decreasing upon microhydration from the point of view of the neutral but do not shift from the point of view of the anion – has been already reported in the authors' last year Nature Chemistry paper on anthracene. What is new here, however, is the fact that the dynamics of the resonances is influenced extremely little upon microhydration. This is surprising, especially in the view of the often-reported suppression of the dissociation channels in various molecules upon microhydration.

2. What is the immediate significance of this advance?
I see two-fold significance: (i) the new effects in the decay dynamics of resonances and (ii) the astrophysical implications outlined by the authors. The latter is valid only if the authors address the technical point below.

3. Technical suggestions
The authors state that the astrophysical models assume that only PAHs containing more than thirty carbon atoms carry the negative charge. This claim is supported by referencing papers 22-25. I have failed to locate this information in these three references (it might be my mistake – these are rather extensive reviews full of information). The authors should provide a brief explanation for this minimum size in the current paper and a more precise reference.

Author's Response to Peer Review Comments:

Dear Editor,
Thank you for passing on the comments. We are delighted that all three referees liked the work. We have made the minor revisions suggested by the reviewers (see below) and have uploaded a version of the manuscript where the changes have been tracked.

Reviewer(s)' Comments to Author:

Reviewer: 1

Recommendation: This paper is publishable subject to minor revisions noted. Further review is not needed.

Comments:
I offer the following suggestions to improve this manuscript in the context of fully supporting its publication in JPCL:

0) The water evaporation process described here is really analogous to dissociative electron attachment which is well-known to produce stable anion from electron attachment. I think that this should be pointed out explicitly to help frame this work for those interested in the formation of anion in the interstellar medium.

Good point – we have added this comment when discussing the evaporation.

1) On page 3, line 33, it should refer to Figure 1 instead of Figure 2.

Good spot – changed.

2) The authors gloss over the description of Figure 2 as if everybody was able to understand them by now. It might be just me making an early descent into senility, but even after seeing these 2D plots multiple times, I really struggle with how to interpret them. This is even worst here since each panel is very small. I understand that they were stacked horizontally to emphasize that the resonances don't change in energy with hydration, but it took me an entire (American-sized) cup of coffee to figure it out. It might be useful to dress these figure a bit to help the non-experts.

Yes, we agree that these are still pretty new, but on the other hand, the reader is referred explicitly to the study in which we have dressed up the 2D spectrum. We have added a quick comment to further encourage the reader to visit that work.

3) Referring to the anion resonances strictly by their symmetry is confusing at times since there are two 2B3g and two 2B2g. Maybe a letter in from of the state? X2Au, a2B1u, etc.

Agreed – We have added a number to resolve the confusion.

4) Some description of the expected interaction of water with the pyrene anion would be useful. I assume that they form weakly interacting clusters on top of the plane of the pyrene, similar to the naphtalene-water in ref 44? In that case, it might be too surprising that the stabilization is similar for all the pyrene anion excited-states (which all have transition dipoles moment in the molecular plane?).

Yes, we do effective say this, but agreed that we could be clearer and have added a sentence highlighting the point about the TDM.

5) In Figure 4, I don't see the loss of 3-4 waters for Py(H2O)4 mentioned in the text. (I squinted and stared really hard).

We state that we see up to 4 waters from Py(H2O)4 and up to 3 from Py(H2O)3. This is quite clear from the figure.

6) As a side comment, the detection of the water loss relies on the dissociation and photodetachment within the same laser pulse which might really understate the importance of this pathway relative to autodetachment/thermionic emission. Using a secondary MS stage to separate to the photofragments, as typically used in ion action spectroscopy, would enable the direct measurement of the branching ratio for the evaporation process.

We cannot agree more and a reflectron mass spectrometer will be implemented (once I find some money behind the sofa ☺).

Reviewer: 2

Recommendation: This paper is publishable subject to minor revisions noted. Further review is not needed.

Comments:
This is another nice 2D photoelectron spectroscopic study from the Verlet group. It is rather similar to their recent Nature chemistry (13/2021/737) opus which applied similar techniques to anthracene and nitrogen substituted anthracene derivative, which also reported similar findings. However, the current work focuses on pyrene and solvated pyrene molecules.
The main findings are that resonances in the solvated pyrene molecules become stabilized by solvation (with respect to electron loss) but have very similar energies relative to the anion ground state regardless of the level of solvation. The results also highlight the possibility of relatively small PAHs acting as negative charge carriers in the interstellar media. The results are sufficiently different to the authors' previous work (pyrenes vs anthracenes), sufficiently novel and definitely interesting enough to warrant publication in JPCLett.
The paper is very accessible/readable and I enjoyed the narrative very much.

There are some minor typos that the authors might want to address, but scientifically I have very little to add, although the authors may want to consider backing up the statements on pages 7/8 regarding the two photon process producing Py-. Have the authors attempted to observe the power scaling of the photon intensity dependence?

We have not done the power-dependence (simply because there is already so much work that goes into these experiments), but genuinely do not believe we need them as there is simply no other explanation for what we see.

Reviewer: 3

Recommendation: This paper is publishable subject to minor revisions noted. Further review is not needed.

Comments:

1. What is the major advance reported in the paper?
This is an experimental study on the photodetachment from the mixed pyrene-water anions. The anions are mass selected and thus the authors have full control on the hydration degree. The main experimental observation – that the resonance energies are decreasing upon microhydration from the point of view of the neutral but do not shift from the point of view of the anion – has been already reported in the authors' last year Nature Chemistry paper on anthracene. What is new here, however, is the fact that the dynamics of the resonances is influenced extremely little upon microhydration. This is surprising, especially in the view of the often-reported suppression of the dissociation channels in various molecules upon microhydration.

2. What is the immediate significance of this advance?
I see two-fold significance: (i) the new effects in the decay dynamics of resonances and (ii) the astrophysical implications outlined by the authors. The latter is valid only if the authors address the technical point below.

3. Technical suggestions
The authors state that the astrophysical models assume that only PAHs containing more than thirty carbon atoms carry the negative charge. This claim is supported by referencing papers 22-25. I have failed to locate this information in these three references (it might be my mistake – these are rather extensive reviews full of information). The authors should provide a brief explanation for this minimum size in the current paper and a more precise reference.

This is in the references suggested though I agree that they are hard to read and extensive. Nevertheless, we have added a statement which has been used to justify the 30 C atoms which is based on a sufficiently large electron affinity (that increases it PAH size).
